# Identification and Visualization of CD8^+^ T Cell Mediated IFN-γ Signaling in Target Cells during an Antiviral Immune Response in the Brain

**DOI:** 10.1371/journal.pone.0023523

**Published:** 2011-08-29

**Authors:** Mariana Puntel, Robert Barrett, Nicholas S. R. Sanderson, Kurt M. Kroeger, Niyati Bondale, Mia Wibowo, Sean Kennedy, Chunyan Liu, Maria G. Castro, Pedro R. Lowenstein

**Affiliations:** Board of Governors' Gene Therapeutics Research Institute, Cedars-Sinai Medical Center, Departments of Medicine and Molecular and Medical Pharmacology, David Geffen School of Medicine, University of California Los Angeles, Los Angeles, California, United States of America; The University of Chicago, United States of America

## Abstract

CD8^+^ T cells infiltrate the brain during an anti-viral immune response. Within the brain CD8^+^ T cells recognize cells expressing target antigens, become activated, and secrete IFNγ. However, there are no methods to recognize individual cells that respond to IFNγ. Using a model that studies the effects of the systemic anti-adenoviral immune response upon brain cells infected with an adenoviral vector in mice, we describe a method that identifies individual cells that respond to IFNγ. To identify individual mouse brain cells that respond to IFNγ we constructed a series of adenoviral vectors that contain a transcriptional response element that is selectively activated by IFNγ signaling, the gamma-activated site (GAS) promoter element; the GAS element drives expression of a transgene, *Cre* recombinase (Ad-GAS-Cre). Upon binding of IFNγ to its receptor, the intracellular signaling cascade activates the GAS promoter, which drives expression of the transgene *Cre* recombinase. We demonstrate that upon activation of a systemic immune response against adenovirus, CD8^+^ T cells infiltrate the brain, interact with target cells, and cause an increase in the number of cells expressing *Cre* recombinase. This method can be used to identify, study, and eventually determine the long term fate of infected brain cells that are specifically targeted by IFNγ. The significance of this method is that it will allow to characterize the networks in the brain that respond to the specific secretion of IFNγ by anti-viral CD8^+^ T cells that infiltrate the brain. This will allow novel insights into the cellular and molecular responses underlying brain immune responses.

## Introduction

Viruses cause infections in the brain that result in acute disease of varying severity associated with clearance of the virus, or in chronic progressive disease associated with virus persistence. CD8^+^ T cells are critical immune effectors for viral clearance [1], [2] [3], [4]; failure of CD8^+^ T cell effector function is associated with persistent infection of humans (i.e., HIV and hepatitis C virus), and mice (i.e., lymphocytic choriomeningitis virus) [5], [6], [7].

The functions of CD8^+^ T cells known to be important for virus clearance include their specificity for target antigens, cytotoxic activity and secretion of effector cytokines, and their capacity to migrate and localize to tissue sites of virus infection [8] [9]. In vivo, effector CD8^+^ T cells establish immunological synapses with target cells; immunological synapses constitute the anatomical substrate that facilitate the functional interactions of T cells with their specific targets [10], [11], [12], [13], [14], [15], [16], [17]. Also, immunological synapses restrict the cytotoxic effects of T cells exclusively to their targets; whether they also act similarly to restrict the effects and diffusion of effector cytokines remains unknown –there is evidence that many cytokines may either be secreted diffusely from T cells, or leak out of the immunological synapse – [18], [19], [20].

In some cases, viral infections in the brain could be limited by noncytolytic mechanisms such as IFN-γ [21]. Noncytolytic clearance of Sindbis virus has been described [22]. http://www.plospathogens.org/article/findArticle.action?author=Burdeinick-Kerr&title=Noncytolytic%20clearance%20of%20Sindbis%20virus%20infection%20from%20neurons%20by%20gamma%20interferon%20is%20dependent%20on%20Jak/STAT%20signaling. To what degree different viruses are controlled in vivo through cytotoxic vs. non-cytolytic mechanisms remains to be determined.

Adenoviral vectors are powerful gene transfer tools for transgene expression in the brain. Adenoviral vectors transduce a variety of brain cells, including astrocytes, and allow long term widespread transgene expression if injected directly and carefully into the brain parenchyma [23]. In the presence of an anti-adenovirus immune response transgene expression and vector genomes are eliminated [17], [23], [24] [25]. The immune response to viral vectors is an important challenge to gene therapy, as killing of transduced cells will counter therapeutic benefits.

Long term gene transfer in humans is limited by preexisting anti-adenoviral immunity, or when animals become immunized against adenovirus, as the adaptive immune response reduces transgene expression [17], [23], [26], [27], [28], [29] [13]. In the context of this interaction IFN-γ becomes polarized at intercellular junctions between T cells and infected astrocytes [13].

Upon IFNγ release and binding to its receptor on target cells, IFNγ signals through the JAK-STAT pathway. This causes phosphorylation of STAT1; dimers of phosphorylated STAT1 translocate to the nucleus where, upon binding to specific IFNγ response elements (known as Gamma Activated Sites or GAS), they activate expression of a large number of IFNγ-inducible genes [30]. Subsequent expression of IFNγ-induced proteins is responsible for the antiviral effects of IFNγ.

To identify brain cells that have been exposed to IFNγ we developed a new detection system. Three adenoviral vectors containing the GAS element driving expression of the transgene *Cre* recombinase were produced. One vector did not contain any transgene in addition to the IFNγ-inducible *Cre* recombinase (Ad-GAS-Cre), a second one contained eGFP under the transcriptional control of a constitutive promoter (Ad-eGFP-GAS-Cre), and the third vector contained the HSV1-TK gene under the control of a constitutive MIE-hCMV promoter (Ad-TK-GAS-Cre). *Cre* recombinase was chosen as a transgene –the enzyme is identified by immunocytochemistry, as it becomes concentrated in the nucleus of target cells-.

To determine the efficiency of our vectors to detect IFNγ release in the brain we injected the reporter adenoviral vectors into the brain, and at a later time, induced a systemic immune response to adenovirus, leading to the infiltration of the brain with antiviral CD8^+^ T cells, close interaction with the infected cells and predicted release of IFN-γ. As we detected expression of *Cre* recombinase, with all three vectors, we conclude that our vectors can be used as reporters to monitor the cellular targets of IFNγ secretion in the brain in vivo, during anti-viral immune responses.

## Materials and Methods

### Ethic statement

All animal work has been conducted according to relevant national and international guidelines. The work described was approved by the Institutional Animal Care and Use Committee at Cedars-Sinai Medical Center and conformed to the policies and procedures of the Cedars-Sinai Medical Center Comparative Medicine Department, and was approved under IACUC protocol number 2090.

### Adenovirus vector constructions

**Figure 1:**
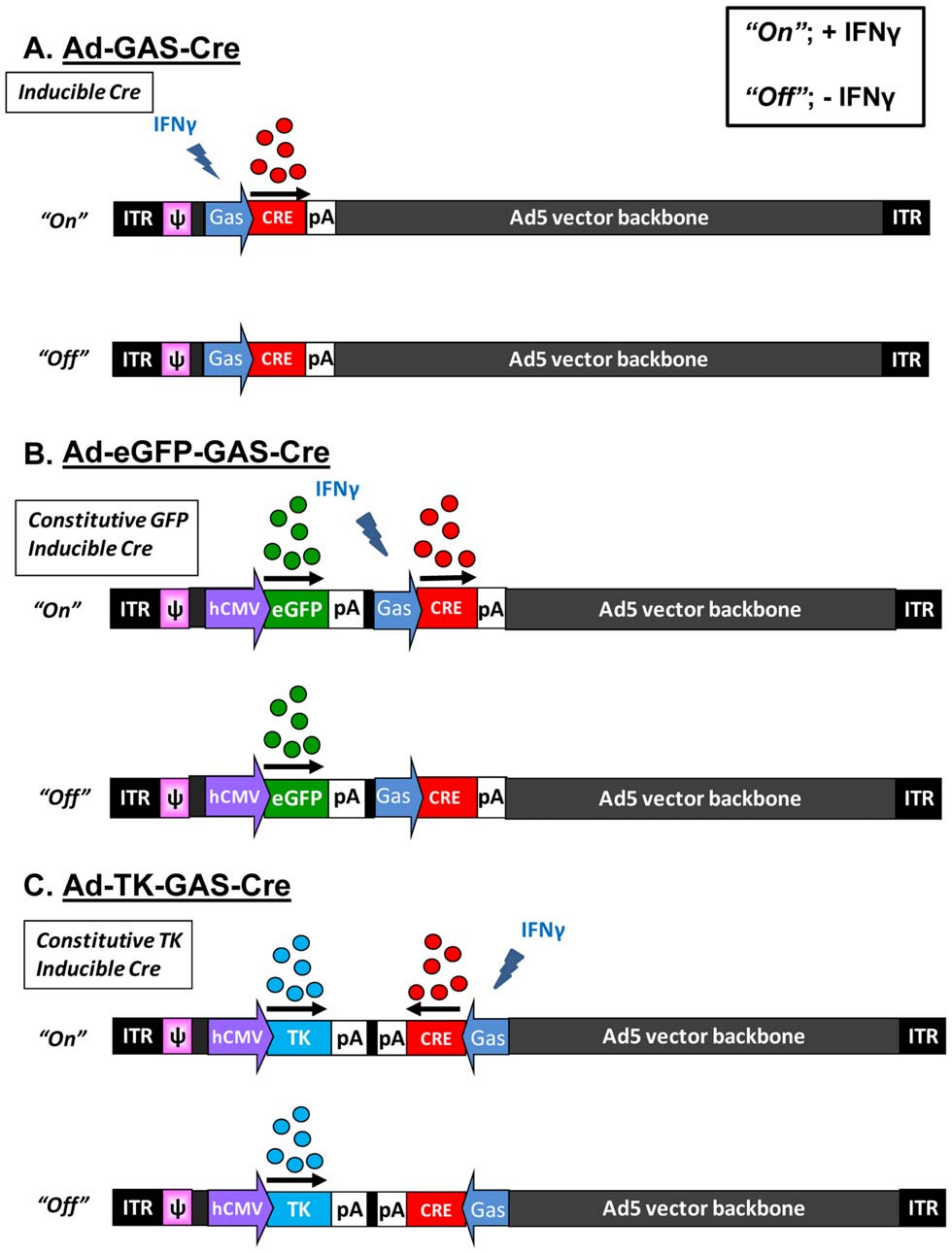
Description of novel viral vectors. Schematic illustration depicting the IFNγ inducible expression of *Cre* recombinase in (**A**) Ad-GAS-Cre; (**B**) Ad-eGFP-GAS-Cre; and (**C**) Ad-TK-GAS-Cre. The figure illustrates that *Cre* recombinase is only expressed in the presence of IFNγ; eGFP and HSV1-TK are expressed constitutively. “On” and “Off” refers to the inducible expression from the IFNγ responsive GAS promoter.

Ad-GAS-Cre, Ad-eGFP-GAS-Cre, and Ad-TK-GAS-Cre are first-generation, replication defective serotype 5 adenovirus with deletions in the E1 and E3 regions. Ad-GAS-Cre is a novel vector constructed to detect IFNγ targeted cells. Ad-GAS-Cre encodes for *Cre* recombinase (a Type I topoisomerase from P1 bacteriophage) driven by an Interferon gamma activated sequence (GAS). Briefly, the GAS element was excised by BamHI digestion from pGAS-Luc (Stratagene, La Jolla) and cloned into pSP72, generating pSP72-GAS-pA (3,362 bp). An extended version of *Cre* recombinase (nls Cre) encoding the nuclear localization signal (nls) from pSV40 large T antigen [31] was PCR amplified with specific primers designed introducing a TATA box in the 5′ extreme and flanking XbaI sites: Cre For 5′-GCTCTAGAGCTATATAATGGCACCCAAGAAGAAGAGGAAGGTGTCC-3′ Cre Rev 5′GCTCTAGAGCCTAATCGCCATCTTGCAGCAGGCGCACCATTGCCCC-3′; and then cloned into the XbaI site of pSP72-GAS-pA, generating pSP72-GAS-Cre (4,411 bp). The GAS-cre cassette was excised from pSP72GAS-Cre by BglII digestion and ligated into pΔE1sp1A (6,409 bp), generating pΔE1sp1A-GAS-Cre (7,387 bp) (**Figure 1A**, **Figure S1**).

Ad-hCMV.eGFP-GAS-Cre (Ad-eGFP-GAS-Cre) is a novel bicistronic adenovirus vector encoding: (i) enhanced green fluorescent protein (eGFP) gene driven by the human cytomegalovirus intermediate-early promoter (hCMV), (ii) GAS-Cre cassette. The pΔE1sp1A-hCMV.eGFP plasmid was generated by cloning the hCMV.eGFP cassette from pAL119.eGFP into the BglII site of pΔE1sp1A-GAS-Cre (**Figure 1B**, **Figure S2**).

Ad-hCMV.TK-GAS-Cre (Ad-TK-GAS-Cre) is a novel bicistronic adenovirus vector encoding: (i) *HSV-1 Thymidine Kinase* gene driven by the human cytomegalovirus intermediate-early promoter (hCMV), (ii) GAS-Cre cassette. The pΔE1sp1A-hCMV.TK plasmid was generated by cloning the hCMV.TK cassette from pAL119TK into the HindIII site of pΔE1sp1A. The GAS-cre cassette was excised from pSP72GAS-Cre by BglII digestion and ligated into pΔE1sp1A-hCMV.TK, generating pΔE1sp1A-hCMV.TK-GAS-Cre (10,329 bp) (**Figure 1C**, **Figure S3**).

Ad-0, Ad-IFNγ [32], Ad-IFNα, and Ad-HPRT have been described elsewhere [33]. First generation vectors were scaled up as described previously [33]. Vector preparations were screened for the presence of replication competent adenovirus and for lipopolysaccharide contamination (Cambrex, East Rutherford, NJ) [33]. Virus preparations used were free from replication competent adenovirus and lipopolysaccharide contamination.

### Animals and surgical procedures

Female B6;129-*Gt(ROSA)26Sor^tm1Sho^*/J (ROSA26) (Jackson Laboratories, Bar Harbor, ME) were injected into the right striatum as previously described with either 1×10^7^ iu of Ad-GAS-Cre, Ad-TK-GAS-Cre, or Ad-eGFP-GAS-Cre. Two weeks later animals were injected systemically (intraperitoneal: ip) with either 5×10^8^ iu of Ad-HPRT in 100 µl of solution or saline. Four weeks after systemic immunization with adenovirus, mice were anesthetized using ketamine (75 mg/kg) and dex-medetomidine (0.5 mg/kg). At experimental endpoints mice were anesthetized and perfused-fixed and brains were processed for immunohistochemistry. Spleens were harvested during anesthesia, before perfusion, and processed for splenocyte isolation as described elsewhere [34]. Spleen cells were stored in liquid nitrogen until used.

C57/Bl6 mice (n = 2) were stereotactically injected bilaterally with 1×10^7^ of Ad-eGFP-GAS-Cre and 21 days later with either Ad-0, Ad-IFNγ, or Ad-IFNα (1×10^7^ iu). Mice were euthanized 14 days later and brain sections were assessed by ICC for *Cre* recombinase. The numbers of *Cre* recombinase immunoreactive cells and eGFP expressing cells were quantified by stereology.

### Immunohistochemistry and immunofluorescence

Coronal brain sections (50 µm thick) were used for immunohistochemistry to detect transgene expression or specific immune cells using methodologies described previously [33]. The primary antibodies used for immunohistochemical staining were the following: rabbit polyclonal anti-*Cre* recombinase (1∶10,000) (Novagen), rabbit polyclonal anti-HSV1-TK (1∶10,000) (produced by us) and rat anti-mouse CD8α (1∶5,000) (Serotec, Raleigh, NC). Appropriate secondary antibodies were used: biotin-conjugated goat anti-rabbit immunoglobulin G (1∶800), biotin-conjugated goat anti-rat immunoglobulin G (1∶800) (DAKO, Glostrup, Denmark), Alexa 488-conjugated goat anti-rabbit (1∶1,000), Alexa 594-conjugated goat anti-rat (1∶1,000), Alexa 647-conjugated goat anti-rat (1∶1,000) (Molecular Probes, Carlsbad, CA).

### Quantification and stereological analysis

The number of labeled cells was quantified using a Zeiss AxioPlan 2 Imaging microscope (Carl Zeiss Microsystems, Thornwood, NY) controlled by a Ludl electronic MAC 5000 XY stage control (Ludl Electronics Products, Hawthorne, NY), and Stereo Investigator software [35]. Data was expressed as an absolute number of positive cells in each anatomical region analyzed. Results were expressed as the mean ±SEM.

### Confocal imaging

Sections were examined using a Leica DMIRE2 confocal microscope (Leica Microsystems) and Confocal Software (Leica Microsystems), as described previously [16].

### ELISPOT assay

As a measure of the cell-mediated immune response to adenovirus we assessed the frequency of IFN-γ-producing T cells using the ELISPOT kit assay (R&D Systems, Minneapolis, MN) according to the manufacturer's instructions. Briefly, splenocytes (1×10^6^ cells/well) were cultured in Millipore MultiScreen-HA plates (coated with anti-IFN-γ antibody) for 24 hours in X-Vivo media (Cambrex, Baltimore, MD) containing 1×10^9^ iu/ml of heat-inactived Ad-HPRT (inactivated at 85°C, for 15 minutes). The wells were then washed and incubated overnight at 4°C with biotinylated anti-IFN-γ detection antibodies (R&D Systems, Minneapolis, MN). Reactions were visualized using streptavidin-alkaline phosphatase, and 5-bromo-4-chromo-3-indolylphosphatase p-toluidine salt, and nitro blue tetrazolium chloride as substrate [34]. The number of spots per 10^6^ splenocytes, which represents the frequency of IFN-γ-producing cells, was counted with the KS ELISPOT automated image analysis system (Zeiss, Jena, Germany). Results were expressed as the mean ±SEM.

### Depletion of circulating CD8^+^ T cells

To determine the optimum timing for the administration of CD8 T cell depletion naïve ROSA26 mice were injected i.p. with 0.5 mg of anti-CD8 depleting antibody once; 3, 7 and 8 days post-depletion two mice were euthanized for the identification of CD8^+^ T cells (CD3^+^, CD4^−^, CD8^+^) by flow cytometry (**Figure S4**). As the circulating levels of CD8^+^ T cells started to recover at 8 days post-injection of the depleting antibody, CD8-depleting antibodies were administered every 5 days, from 2 weeks post-systemic immunization (i.e., day 28, to euthanasia on day 42.

### Statistical analysis

Data were analyzed by using a one way analysis of variance (ANOVA), followed by Tukey's test, or paired T-test; results were expressed as the mean ± SEM. A ‘p’ value <0.05 was considered the cut off for significance.

## Results and Discussion

### Development of a novel system to detect IFNγ signaling in the brain *in vivo*

**Figure 2:**
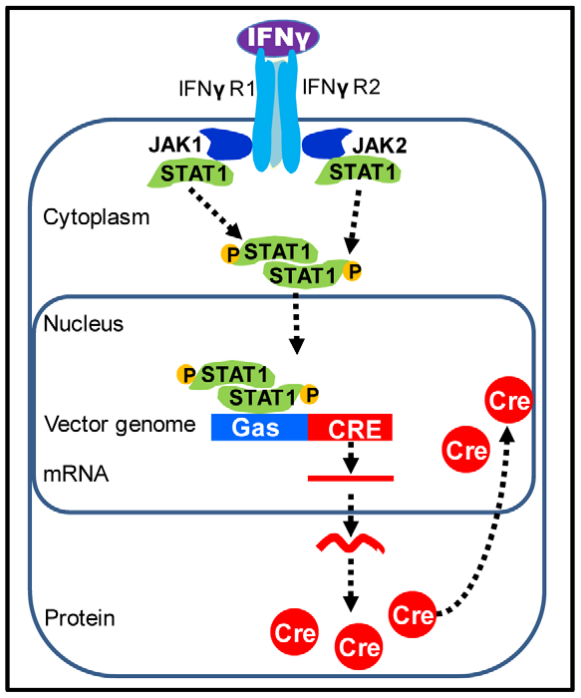
Schematic of the IFNγ signaling pathway, and the use of GAS-Cre as a reporter of IFNγ signaling. Schematic of IFNγ signaling through the JAK/STAT1 pathway. Binding of IFNγ to its receptor results in activation of JAKs, which phosphorylate cytoplasmic monomers of STAT1. Phosphorylated STAT1 dimerizes, translocates to the nucleus, and activates transcription from gene promoters containing Gamma Activated Sites (GAS). *Cre* recombinase translocates to the nucleus due to the presence of a nuclear localization signal.

To identify and visualize IFN-γ secretion onto target cells during an active immune response against adenovirus, we generated three novel first generation adenoviral vectors encoding *Cre* recombinase under a promoter (GAS) that is inducible upon IFNγ binding and stimulation of the IFNγ receptor and its specific downstream signaling (**Figures 1**, **2**). The schematic genetic structure of all three vectors, Ad-GAS-Cre, Ad-TK-GAS-Cre, or Ad-eGFP-GAS-Cre, is shown in **Figure 1**.

Ad-GAS-Cre was constructed first; upon stimulation with IFNγ *Cre* recombinase expression was detected demonstrating the functionality of the GAS-Cre IFNγ inducible expression cassette within first generation adenoviral vectors. However, Ad-GAS-Cre does not allow to detect transduction of target cells in the absence of IFNγ. Thus, to identify transduced cells during an anti-adenoviral immune response, and to be able to do so independently of the presence of IFNγ we constructed two additional vectors, Ad-eGFP-GAS-Cre (**Figure 1B**), and Ad-TK-GAS-Cre (**Figure 1C**). Ad-EGFP-Cre encodes the fluorescent protein *enhanced green fluorescent protein*, and Ad-TK-GAS-Cre the HSV-1 Thymidine Kinase; expression of eGFP and HSV1-TK are under the control of a constitutive promoter. Vector-mediated detection of IFNγ signaling in target cells is illustrated in **Figure 2**.

### Transient expression of *Cre* recombinase at early time points after first generation adenoviral vectors administration

**Figure 3:**
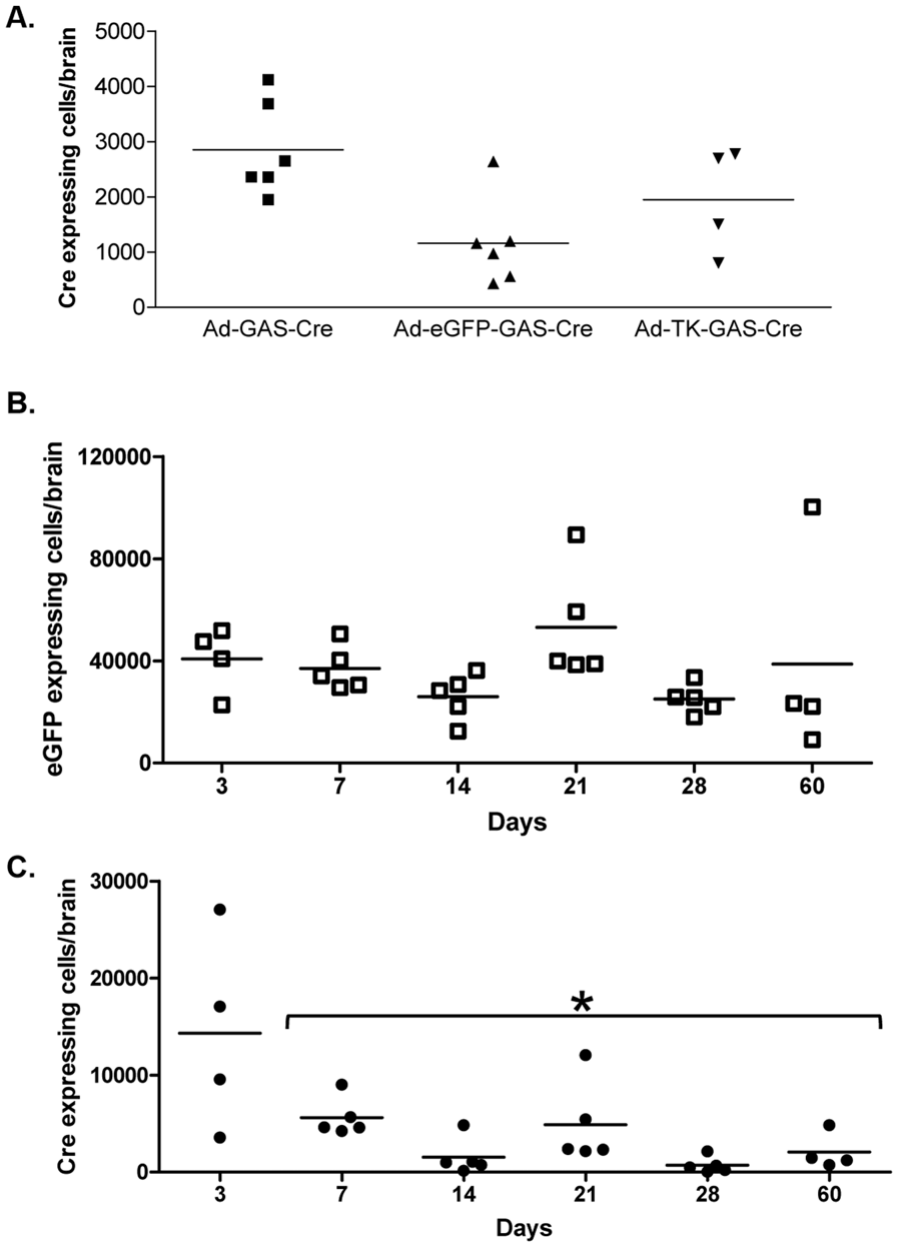
Time course of basal unstimulated *Cre* recombinase expression following the injection of adenoviral vectors expressing *Cre* recombinase. **A.** Low levels of basal (unstimulated) expression of *Cre* recombinase was detected 3 days following the intrastriatal injection of Ad-GAS-Cre, Ad-eGFP-GAS-Cre, and Ad-TK-GAS-Cre, in animals that had not been immunized against adenovirus. To determine the time course of basal *Cre* recombinase expression mice (n = 2–3/time point) were stereotactically injected bilaterally with 1×10^7^ iu of Ad-eGFP-GAS-Cre. Mice were euthanized 3, 7, 14, 21, 28, and 60 days later and brain sections were assessed for eGFP expression (**B**), and by ICC for *Cre* recombinase (**C**). Numbers of eGFP expressing cells and *Cre* recombinase immunoreactive cells were quantified by stereology. Note that eGFP expression remained stable over time, and basal *Cre* recombinase expression was highest at 3 days post-vector delivery, and decreased thereafter. As minimum basal levels were reached at 14 days post-vector delivery, and remained low at 28 and 60 days post-injections, these time points were selected for further studies (i.e., animals in further experiments were injected into the brain at Day 0, immunized systemically against adenoviral vectors on Day 14, and analyzed at 2 and 4 weeks post-immunization). * p<0.05 comparison of all time points, one-way ANOVA followed by Tukey's test.

To determine the existence of any basal expression from our vectors in the absence of a systemic immune response C57/Bl6 mice were intracranially injected with all three viruses. At 3 days after injection we detected *Cre* recombinase expression from all vectors (**Figure 3A**). To determine whether early *Cre* recombinase expression is stable or transient C57/Bl6 mice were stereotactically injected bilaterally in the brain striatum with Ad-eGFP-GAS-Cre and euthanized 3, 7, 14, 21, 28, and 60 days after injection. Quantification of eGFP expression, an indication of adenovirus transduction, was stable over 60 days (**Figure 3B**). Importantly, expression of *Cre* recombinase was reduced over time (**Figure 3C**), indicating that the early expression is transient. As *Cre* recombinase expression reached basal levels at 14 days post-injection, and remained low until 60 days post-injection, it was decided that in experiments to test the effect of a systemic immune response, animals would be immunized at 14 days post-delivery of vectors into the brain, and then sacrificed at either 2 or 4 weeks later to determine whether the systemic immune response was able to induce *Cre* recombinase expression. Administration of systemic IFNγ or IFNα through adenoviral vectors did not increase *Cre* recombinase expression in the brain (data not shown).

### *Cre* recombinase is expressed in the brains of mice during an adenovirus specifc systemic immune response

**Figure 4:**
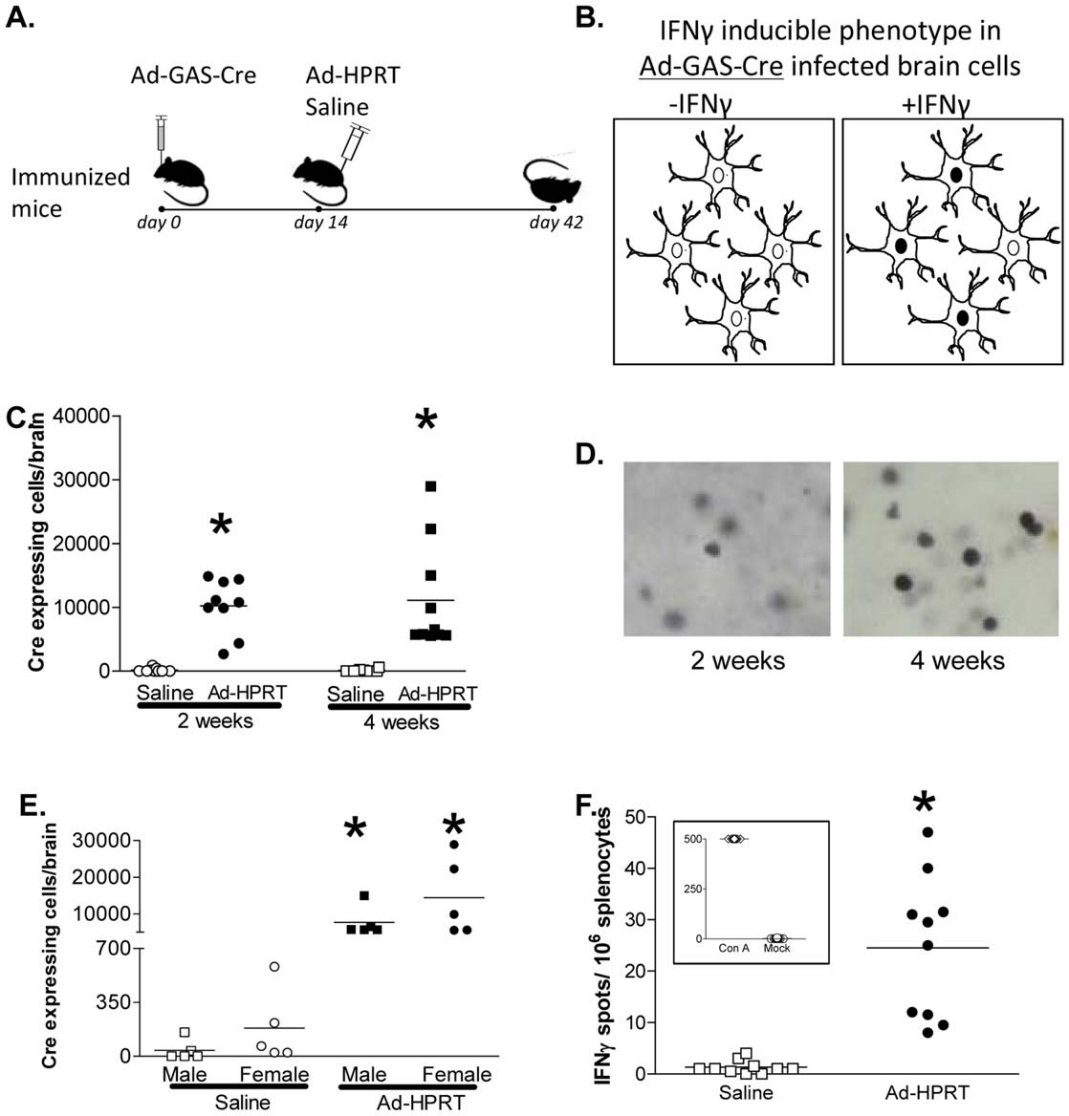
Systemic immunization against adenovirus induces *Cre* recombinase expression in mice injected with Ad-GAS-Cre. **A.** ROSA26 mice (n = 5) were stereotactically injected in the striatum with Ad-GAS-Cre, immunized i.p. 14 days later mice with Ad-HPRT or saline; and euthanized 2 or 4 weeks after immunization; brain sections were assessed by ICC for *Cre* recombinase. **B.** Diagram depicting the paradigm for the IFNγ inducible phenotype of Ad-GAS-Cre infected brain cells in our model; i.e., systemic immunization causes nuclear localization of the transgene *Cre* recombinase (illustrated as black nuclei upon availability of IFNγ). **C.** *Cre* recombinase immunoreactivity is increased in striatal cells at 2 and 4 weeks post-systemic anti-adenoviral immunization; *, p<0.05 versus saline, by paired T-test. **D.** Representative micrographs illustrate striatal cells expressing nuclear *Cre* recombinase at 2 weeks or 4 weeks post- immunization; **E.** When discriminated by sex, no significant differences were found in the number of *Cre* recombinase expressing cells in the brain of males compared to females in either sham or immunized mice; *, p<0.05 versus saline, by paired T-test. **F.** Total splenocytes were tested to determine the frequency of IFNγ secreting lymphocytes specific for adenovirus antigens using ELISPOT assay. Concavalin A (ConA) was used as a control mitogen. *, p<0.05 versus saline, by paired T-test.

ROSA26 mice were injected into the right striatum with 1×10^7^ iu of Ad-Gas.Cre (**Figure 4**). Two weeks later animals were injected ip with either 5×10^8^ iu of Ad-HPRT or saline. Two or four weeks after systemic immunization with adenovirus, mice were euthanized, perfused fixed, and 50 µm thick brain sections were analyzed for expression of *Cre* recombinase. To confirm the efficacy of the immunization, total splenocytes were analyzed for the frequency of IFNγ-secreting immune cells by IFNγ ELISPOT in response to stimulation with adenovirus (**Figure 4F**). At 2 and 4 weeks post immunization the levels of *Cre* recombinase expression in the brain were elevated (**Figure 4C, D, E**). There was no difference in the increase of *Cre* recombinase expression between males and females (**Figure 4E**). These data suggest that IFNγ release in the brain following systemic immunization against adenovirus, mediates an increase in the expression of *Cre* recombinase. ROSA26 mice were utilized to determine whether *Cre* recombinase expression would remove the STOP sequence within the endogenous ROSA26 locus and activate expression of the enzyme β-galactosidase. However, we never were able to detect expression of β-galactosidase. This suggests that either the GAS element alone does not produce sufficient levels of *Cre* recombinase expression to achieve the excision of the STOP sequence, or that the target cells' physiology is affected by the immune system, in such a way that proper gene expression becomes impaired.

### Ad-eGFP-GAS-Cre as a tool to visualize the interactions between CD8^+^ T cells and target brain cells

**Figure 5:**
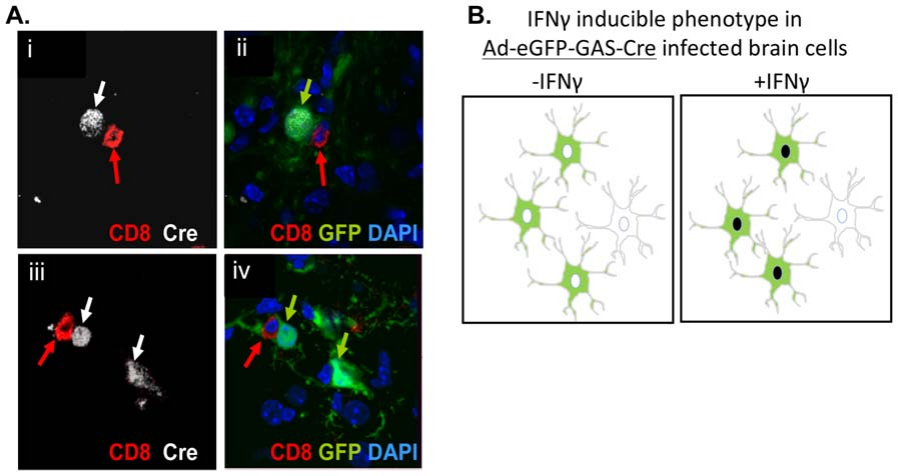
Systemic immunization against adenovirus induces *Cre* recombinase expression in brain cells infected with Ad-eGFP-GAS-Cre. ROSA26 mice (n = 5) were stereotactically injected in the brain with Ad-eGFP.GAS-Cre, immunized i.p. 14 days later with Ad-HPRT or saline, and euthanized 4 weeks post-immunization; brain sections were assessed by ICC for *Cre* recombinase and CD8α **A.** Representative micrographs illustrate cells expressing *Cre* recombinase (grey), eGFP (green), and CD8+ T cells (red) in the striatum of immunized mice. Notice that in some cases close apposition between T cells and infected cells expressing *Cre* recombinase was detected, while other cells expressing *Cre* recombinase did not have an associated T cell, indicating the likely diffusion of IFNγ throughout the tissue; **B.** Diagram depicting the paradigm for the IFNγ inducible phenotype of Ad-eGFP-GAS-Cre infected brain cells in our model; i.e., systemic immunization causes nuclear localization of the transgene *Cre* recombinase (illustrated as dark nuclei upon availability of IFNγ, within green cells expressing eGFP).

Ad-eGFP-GAS-Cre (1×10^7^ iu) was injected into the right striatum of ROSA26 mice (**Figure 5**). Two weeks later animals were injected systemically with either 5×10^8^ iu of Ad-HPRT or saline. Four weeks after systemic immunization with Ad-HPRT, mice were euthanized and brain sections were analyzed for the expression of *Cre* recombinase and CD8α. Close interactions between CD8^+^ T cells and infected brain cells, and between CD8^+^ T and brain cells expressing *Cre* recombinase, were detected in immunized animals (**Figure 5A**). No CD8^+^ T cells, or *Cre* recombinase expression were detected in saline immunized animals (data not shown). As morphological changes were detected in infected cells, which were attributed to eGFP, we sought to study the detailed responses to systemic immunization utilizing a different transgene, namely HSV1-TK.

### IFNγ signaling is increased during the elimination of virally infected cells in the brain following the systemic immunization against adenovirus

**Figure 6:**
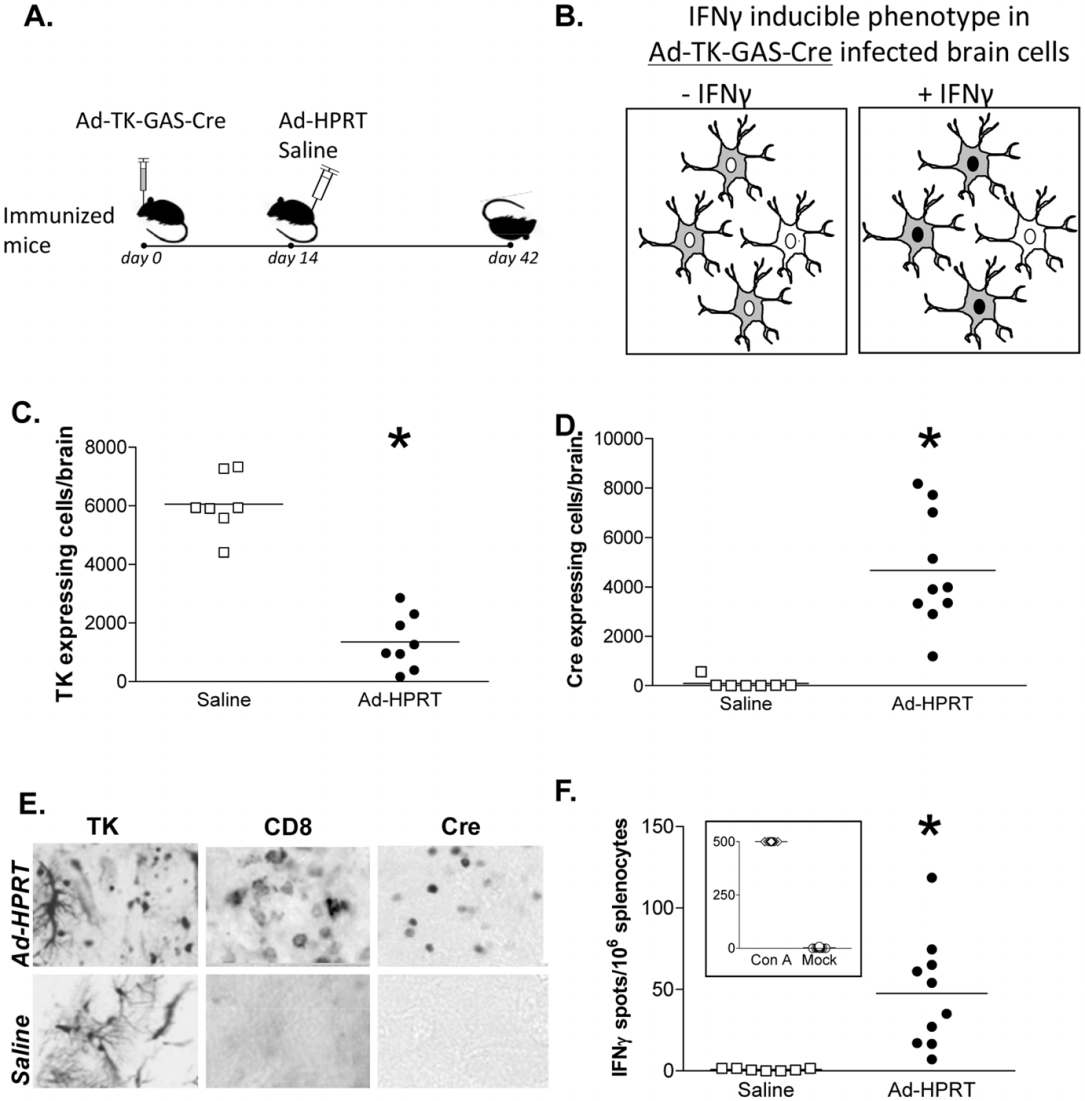
Systemic immunization against adenovirus induces *Cre* recombinase expression in brain cells infected with Ad-TK-GAS-Cre. **A.** ROSA26 mice (n = 5) were stereotactically injected in the brain with Ad-TK-GAS-Cre, immunized i.p. 14 days later with Ad-HPRT or saline, and euthanized 4 weeks after immunization. **B.** Diagram depicting the paradigm for the IFNγ inducible phenotype of Ad-TK-GAS-Cre infected brain cells in our model; i.e., systemic immunization causes nuclear localization of the transgene *Cre* recombinase (illustrated as dark nuclei upon availability of IFNγ, within grey cells expressing HSV1-TK). **C.** HSV1-TK immunoreactivity was used to quantitate levels of viral transduction in the brain, and illustrates the decrease in HSV1-TK transgene expression upon immunization. **D.** *Cre* recombinase immunoreactivity was used to quantitate the level of IFNγ dependent signaling during the anti-adenoviral systemic immune response. **E.** Micrographs depict cells expressing HSV1-TK (left panel), CD8^+^ T cells (middle panel), and *Cre* recombinase (right panel) staining in representative striatal sections from saline treated or mice immunized with Ad-HPRT. **F.** Total splenocytes were assessed for the frequency of IFNγ secreting lymphocytes specific for adenovirus antigens using ELISPOT assay. Concavalin A (ConA) was used as a control mitogen. *, p<0.05 versus saline, by paired T- test.

ROSA26 mice were stereotactically injected in the brain with Ad-TK-GAS-Cre, immunized 2 weeks later mice with Ad-HPRT or saline (ip), and euthanized 4 weeks after immunization (**Figure 6A**). Serial brain sections were analyzed for the expression of *Cre* recombinase, HSV1-TK and CD8α. The number of virally transduced cells expressing HSV1-TK decreased 4 fold (**Figure 6C**). The number of *Cre* recombinase expressing cells increased significantly (**Figure 6D**; shown schematically in **Figure 6B**). CD8^+^ T cell infiltration in the brain parenchyma was detected only in adenovirus-immunized animals (**Figure 6E**). Increased frequency of IFNγ secreting lymphocytes specific for adenovirus antigens, and detected using an ELISPOT assay, was detected among total splenocytes (**Figure 6F**). Our results show that the systemic adenovirus immune response induces abundant CD8^+^ T cell infiltration of the brain, and an increase in the expression of *Cre* recombinase from an IFNγ sensitive promoter, suggesting the presence of elevated IFNγ signaling during the loss of HSV1-TK expression.

### CD8^+^ T cells are necessary for induction of IFNγ-inducible *Cre* recombinase expression *in vivo*, during the loss HSV1-TK expression from adenoviral vectors

**Figure 7:**
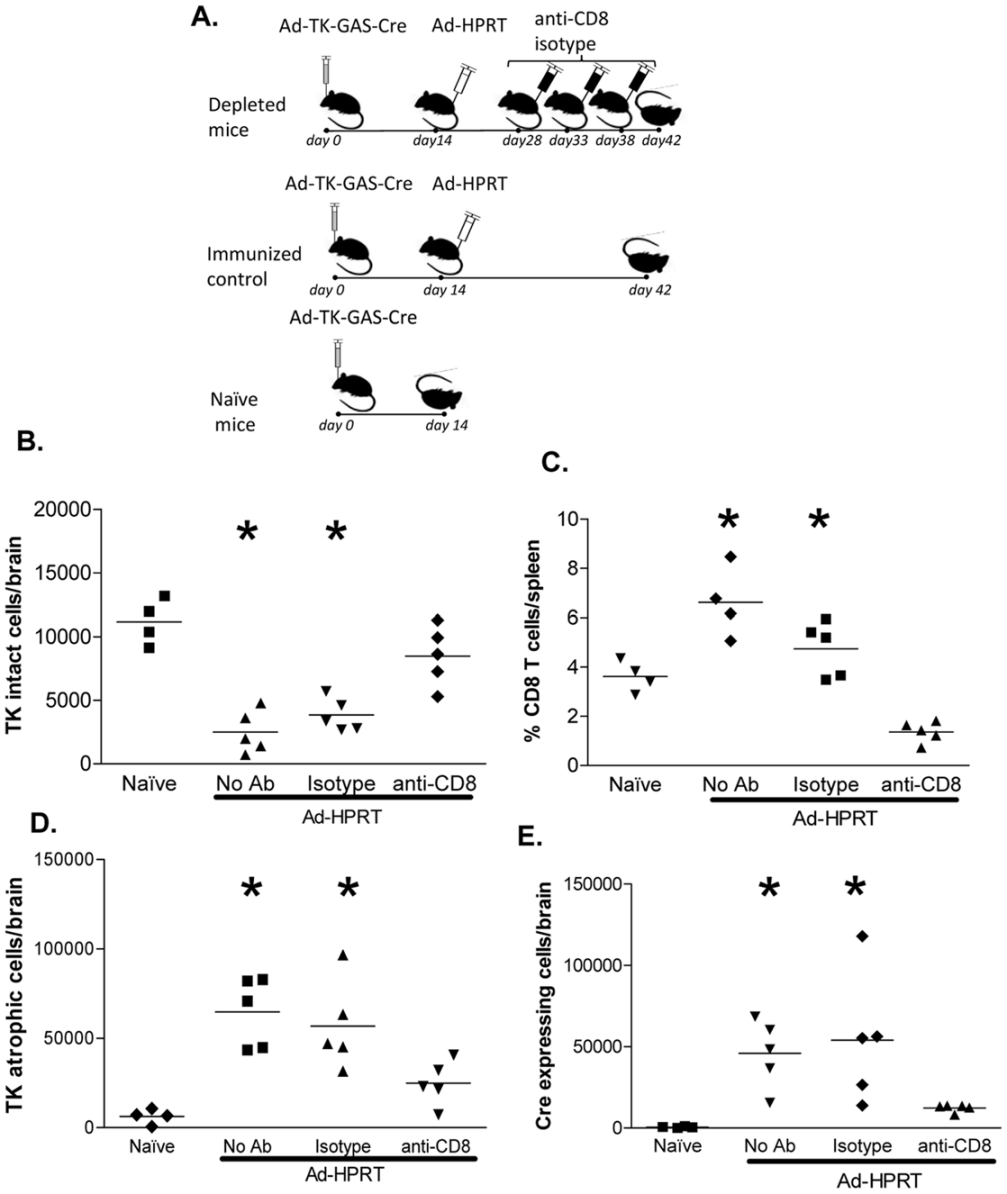
CD8^+^ T cells are necessary to induce IFNγ-dependent *Cre* recombinase expression following the systemic immunization against adenoviral vector. **A.** ROSA26 mice were stereotactically injected in the striatum with Ad-TK-GAS-Cre, immunized i.p. 14 days later with Ad-HPRT. This was followed by the ip injection of 0.5 mg of anti-CD8 depleting antibody (or isotype control, or no antibody) every 5 days until euthanasia, which was performed at 4 weeks after immunization. Brain sections were assessed by ICC for *Cre* recombinase and HSV1-TK. **B.** HSV1-TK immunoreactivity was used to quantitate the levels of morphologically intact virally transduced cells; note that the number of morphologically intact HSV1-TK expressing cells is reduced upon immunization, and this effect is inhibited by the depletion of circulating CD8^+^ T cells. **C.** Flow cytometry was used to determine the percentage of CD8^+^ T cells (CD3^+^CD4^−^CD8^+^) in the spleen of mice; note that the increase of adenovirus-specific CD8^+^ T cells caused by systemic anti-adenovirus immunization is reduced by treatment with CD8-depleting antibodies. **D.** HSV1-TK immunoreactivity was used to quantitate the levels of morphologically atrophic virally transduced cells in the brain –atrophic cells were characterized by the lack of cellular processes, and mainly consisted of cell bodies-; **E.** *Cre* recombinase immunoreactivity was used to quantitate the level of IFNγ-dependent signaling in the brain during the anti-adenoviral immune response; note that *Cre* recombinase expression is increased upon immunization, but blocked by the treatment of animals with CD8-depleting antibodies; *, p<0.05 versus naïve or CD8-depleted mice by one-way ANOVA followed by Tukey's test.

To determine the role of CD8^+^ T cells in stimulating IFNγ-inducible *Cre* recombinase during a systemic anti-adenoviral immune response, we depleted CD8^+^ T cells from animals that were systemically immunized against adenovirus; CD8^+^ T cell was achieved using a specific depleting antibody (set up experiment is illustrated in **Figure S4**). ROSA26 mice were stereotactically injected in the brain with Ad-TK-GAS-Cre, 2 weeks later immunized ip with Ad-HPRT. One group of animals was then injected ip injection of anti-CD8 depleting antibody (or isotype control) two weeks post-immunization, and followed with additional injections of depeleting antibodies every 5 days until euthanasia (**Figure 7A**). Brain sections were analyzed for the expression of *Cre* recombinase and HSV1-TK. The number of HSV1-TK expressing cells decreased significantly in immunized animals treated either with no antibodies, or isotype control, while no decrease was observed in animals treated with the anti-CD8 depleting antibody (**Figure 7B**). Flow cytometry analysis revealed effective reduction of CD8^+^ T cells (CD3^+^CD4^−^CD8^+^) in the spleens of mice after treatment with anti-CD8 depleting antibody; and no difference was detected between groups of animals which had been immunized and either treated with isotype control antibody or none (**Figure 7C**). We also determined the number of cells in which we could still detect HSV1-TK, but were otherwise atrophied, as they were devoid of normal cellular processes shown by cells in non-immunized animals (**Figure 7D**); In parallel, the number of *Cre* recombinase expressing cells increased in immunized animals that were injected with either isotype antibody or no antibody, but increased much less in immunized animals depleted of CD8^+^ T cells (**Figure 7E**). These results suggest that IFNγ induces expression of *Cre* recombinase and that this nuclear expression remains detectable in cells otherwise undergoing atrophy in response to the immune attack, and that CD8^+^ T cells are the main producers of IFNγ in the brain during the antiviral immune response, and thus, directly responsible for the activation of *Cre* recombinase expression in target cells.

### Conclusions

In order to fully understand the cellular and molecular dynamics of the brain immune response, it is important to be able to determine which target cells are influenced during the brain infiltration with CD8^+^ T cells, as these aim to clear the brain from virally infected cells. To determine which cells in the brain respond to IFNγ secreted from T cells upon activation, we developed a series of viral vectors encoding an IFNγ responding element. Using these vectors we demonstrate that widespread IFNγ signaling does indeed occur, as it can be effectively detected in a large amount of target cells during immune-mediated elimination of adenovirally infected cells. The presence of large numbers of *Cre* recombinase expressing cells during the peak of the brain infiltration with anti-viral T cells suggests a large amount of IFNγ activity. The atrophic nature of remaining cells expressing *Cre* recombinase and HSV1-TK, together with their inability of expressing β-galactosidase, suggests that brain cells targeted by the immune system remain with a highly compromised viability. Together with many other data demonstrating the loss of transduced cells in this and similar paradigms, suggests that the immune response is able to eliminate transduced cells from the brain, or seriously damage their physiology. Our data demonstrate that IFNγ signaling occurs in target cells during the brain antiviral immune responses. Our novel method could be used further to study the *in vivo* dynamics of IFNγ secretion and signaling onto target cells in a number of experimental paradigms of neuroimmune pathology.

## Supporting Information

Figure S1 Cloning strategy illustrating the molecular construction of pΔE1sp1A-GAS-Cre (i) Luc gene was cut from pGAS-Luc with XbaI, then pGAS was cut with BamHI and cloned into the BglII site of pSP72BglII to make pSP72-GAS-PolyA; (ii) nlsCre was cut from pGemTATACre with XbaI and cloned into the XbaI site of pSP72-GAS-PolyA to make pSP72-GAS-Cre; (iii) GAS-Cre cassette was cut from pSP72-GAS-Cre with BglII and cloned into the BglII site of pΔE1sp1A to make pΔE1sp1A-GAS-Cre, which was used to construct Ad-GAS-Cre.
(TIF)

Figure S2 Cloning strategy illustrating the molecular development of pΔE1sp1A-hCMVEGFP-GAS-Cre adenovirus genome plasmids. (i) The hCMV eGFP polyA cassette was excised from pAL119eGFP (8,178 bp) by HindIII digestion, extremes were blunted and cloned into EcoRV site of pDE1sp1A, generating pΔE1sp1A-hCMV.eGFP (7,923 bp). (ii) The GAS-Cre-polyA cassette from pSp72.GAS-polyA (3,374 bp) was excised with BglII and cloned into the BglII site of pΔE1sp1A.hCMV.eGFP, generating pΔE1sp1A-hCMV.eGFP-GAS-nlsCre (9,969 bp), which was used to construct Ad-eGFP-GAS-Cre.
(TIF)

Figure S3 Cloning strategy illustrating the molecular construction of pΔE1sp1A-hCMVTK-GAS-Cre adenovirus genome plasmids. The GAS-Cre cassette was cut from pSP72-GAS-Cre with BglII and cloned into the BglII site of pΔE1sp1A –hCMVTK to make pΔE1sp1A-hCMVTK-GAS-Cre, which was used to construct Ad-TK-GAS-Cre.
(TIF)

Figure S4 Testing of the CD8^+^ T cell depletion paradigm. A. Naïve ROSA26 mice were injected i.p. with 0.5 mg of anti-CD8 depleting antibody once. 3, 7 and 8 days post-depletion two mice were euthanized for the identification of CD8+ T cells (CD3^+^, CD4^−^, CD8^+^) by flow cytometry, shown in **B** in the spleen, and in **C**, in the cervical draining lymph nodes. As the number of CD8+ T cells started to recover at 8 days post-injection of the depleting antibody, we injected depleting antibody every 5 days.
(TIF)
