# Supplementary figures and images for: Identification and Visualization of CD8+ T Cell Mediated IFN-γ Signaling in Target Cells during an Antiviral Immune Response in the Brain
Source: PLoS One. 2011 Aug 29;6(8):e23523. doi: 10.1371/journal.pone.0023523 (PMC3163574; doi:10.1371/journal.pone.0023523)

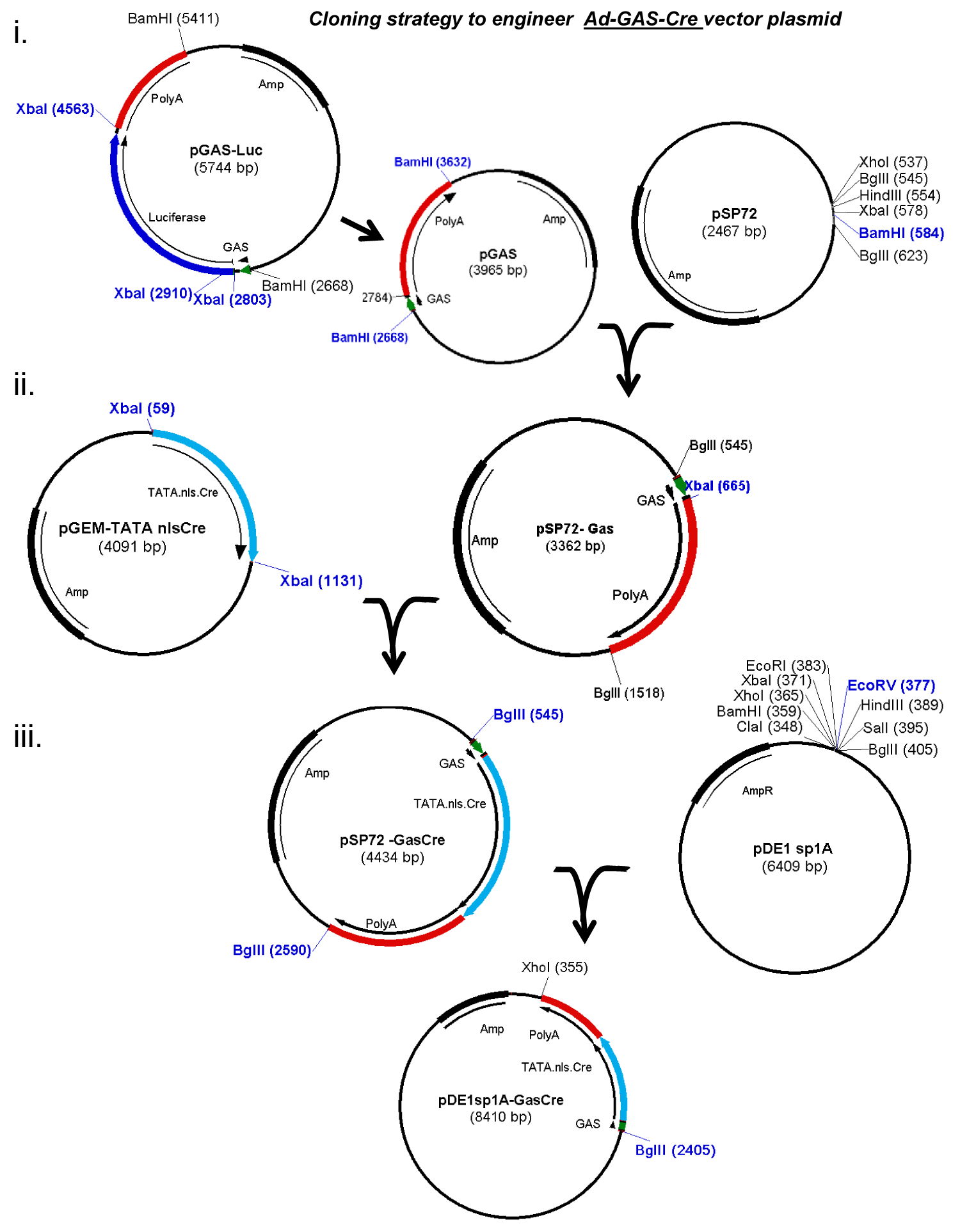

Supplement: Figure S1 — Cloning strategy illustrating the molecular construction of pΔE1sp1A-GAS-Cre (i) Luc gene was cut from pGAS-Luc with XbaI, then pGAS was cut with BamHI and cloned into the BglII site of pSP72BglII to make pSP72-GAS-PolyA; (ii) nlsCre was cut from pGemTATACre with XbaI and cloned into the XbaI site of pSP72-GAS-PolyA to make pSP72-GAS-Cre; (iii) GAS-Cre cassette was cut from pSP72-GAS-Cre with BglII and cloned into the BglII site of pΔE1sp1A to make pΔE1sp1A-GAS-Cre, which was used to construct Ad-GAS-Cre. (TIF) [file pone.0023523.s001.tif]

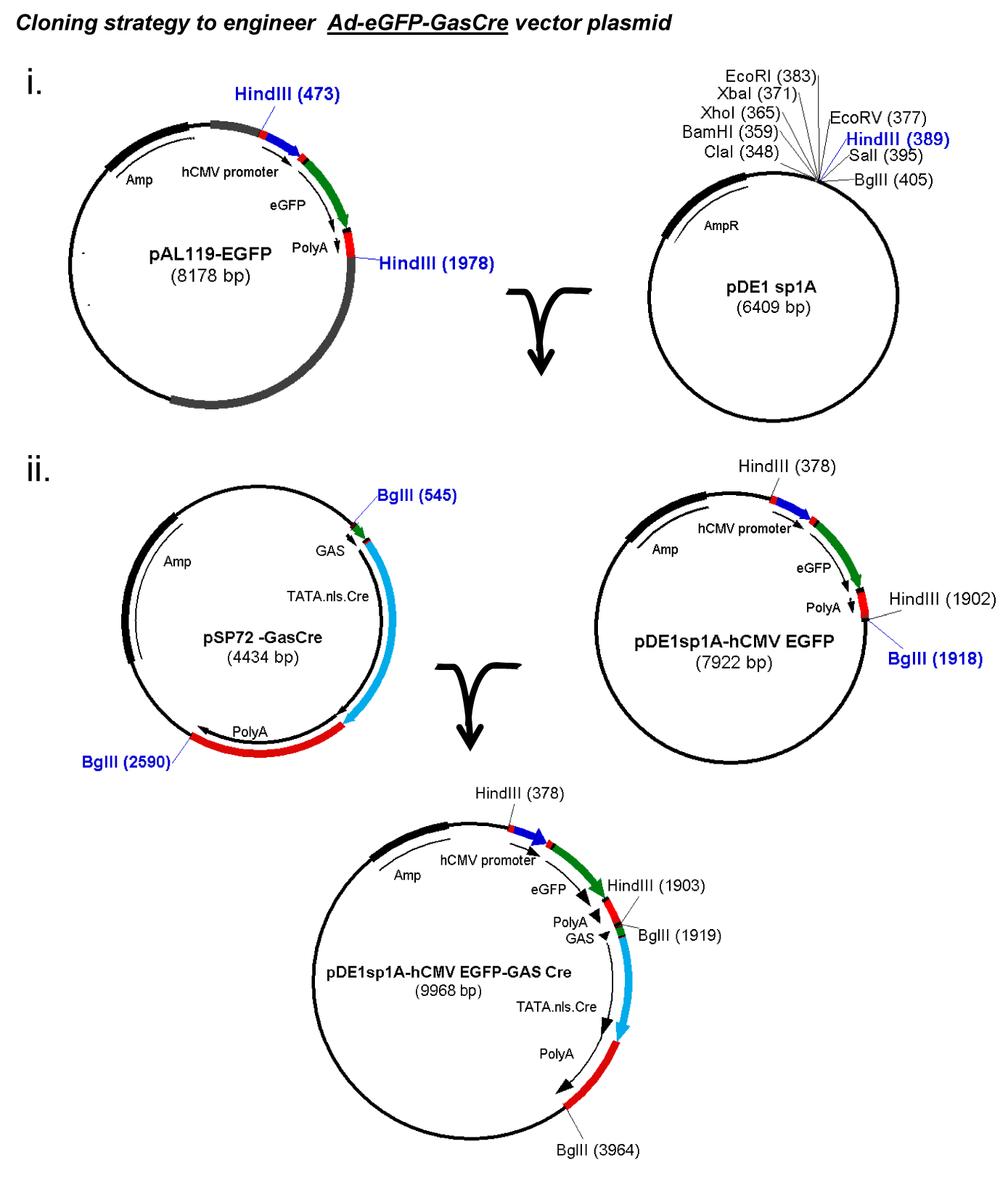

Supplement: Figure S2 — Cloning strategy illustrating the molecular development of pΔE1sp1A-hCMVEGFP-GAS-Cre adenovirus genome plasmids. (i) The hCMV eGFP polyA cassette was excised from pAL119eGFP (8,178 bp) by HindIII digestion, extremes were blunted and cloned into EcoRV site of pDE1sp1A, generating pΔE1sp1A-hCMV.eGFP (7,923 bp). (ii) The GAS-Cre-polyA cassette from pSp72.GAS-polyA (3,374 bp) was excised with BglII and cloned into the BglII site of pΔE1sp1A.hCMV.eGFP, generating pΔE1sp1A-hCMV.eGFP-GAS-nlsCre (9,969 bp), which was used to construct Ad-eGFP-GAS-Cre. (TIF) [file pone.0023523.s002.tif]

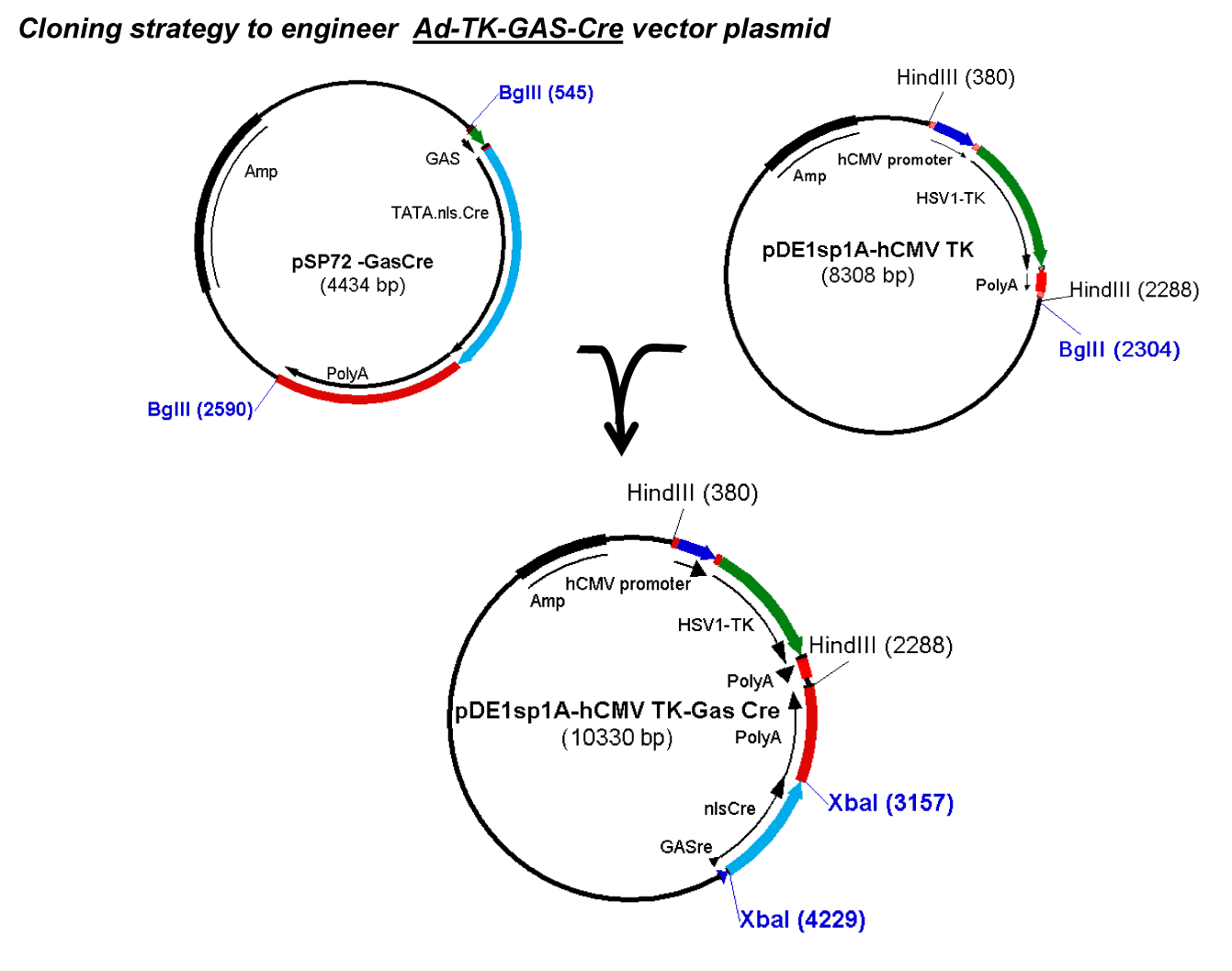

Supplement: Figure S3 — Cloning strategy illustrating the molecular construction of pΔE1sp1A-hCMVTK-GAS-Cre adenovirus genome plasmids. The GAS-Cre cassette was cut from pSP72-GAS-Cre with BglII and cloned into the BglII site of pΔE1sp1A –hCMVTK to make pΔE1sp1A-hCMVTK-GAS-Cre, which was used to construct Ad-TK-GAS-Cre. (TIF) [file pone.0023523.s003.tif]

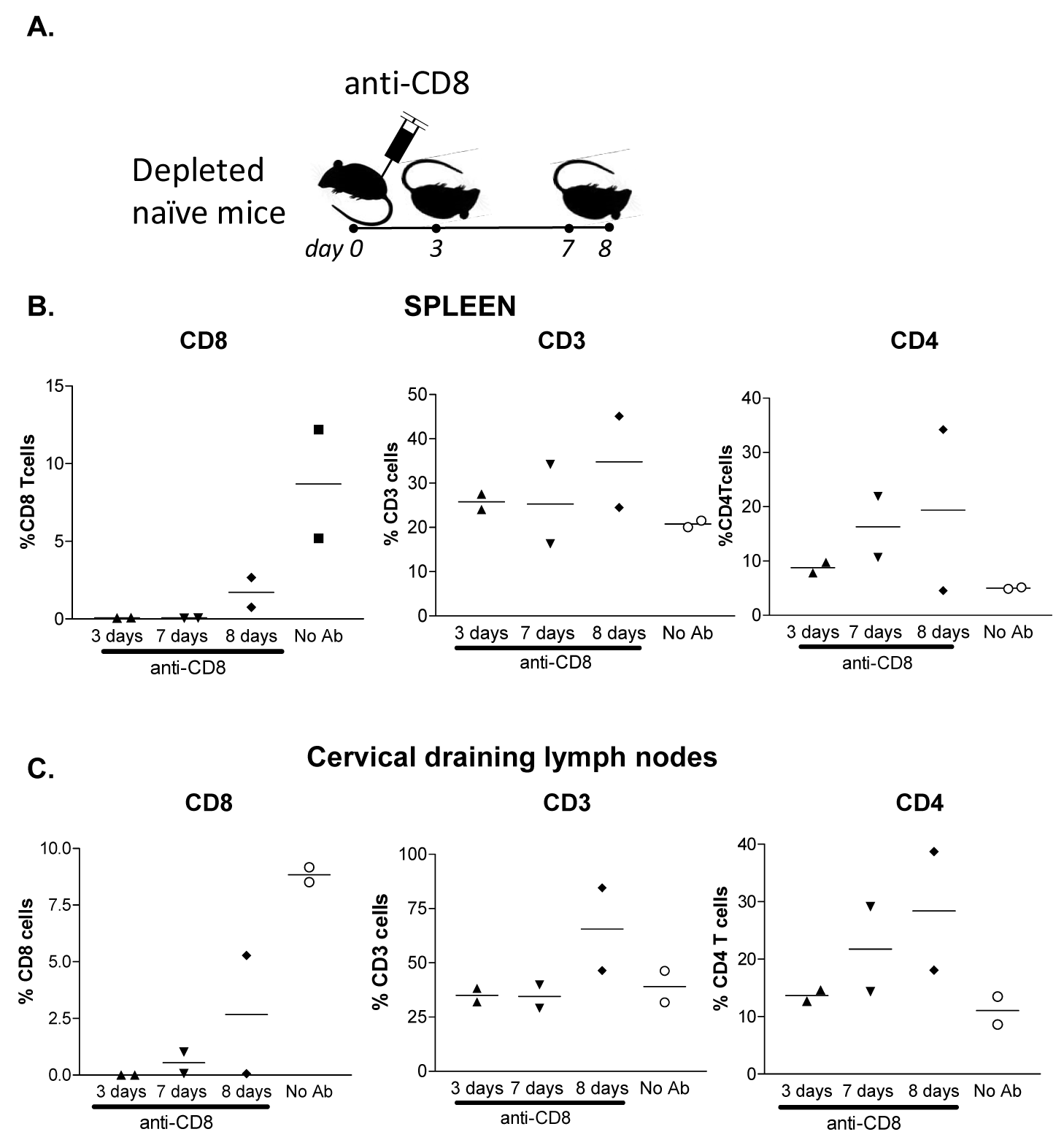

Supplement: Figure S4 — Testing of the CD8+ T cell depletion paradigm. A. Naïve ROSA26 mice were injected i.p. with 0.5 mg of anti-CD8 depleting antibody once. 3, 7 and 8 days post-depletion two mice were euthanized for the identification of CD8+ T cells (CD3+, CD4−, CD8+) by flow cytometry, shown in B in the spleen, and in C, in the cervical draining lymph nodes. As the number of CD8+ T cells started to recover at 8 days post-injection of the depleting antibody, we injected depleting antibody every 5 days. (TIF) [file pone.0023523.s004.tif]
